# C1q-CD44 interactions regulate microglial phagocytosis, proliferation, and migration

**DOI:** 10.21203/rs.3.rs-7769713/v1

**Published:** 2025-10-23

**Authors:** Pooja S. Sakthivel, Alyssa J. Villegas, Anita Lakatos, Meghana Kaipa, Julian M. Lopez, Ashley Ling, Zeina H. Elrachid, Josh Karam, Aileen J. Anderson

**Affiliations:** University of California; University of California; University of California; University of California; University of California; University of California; University of California; University of California; University of California

## Abstract

Microglia, the immune cells of the central nervous system (CNS), quickly respond to neurodegeneration by proliferating and migrating to areas of disease, phagocytosing debris, and releasing cytokines to initiate inflammation. Critically, the mechanisms underlying these microglial functions remain only partly understood. One molecular regulator of interest is complement protein C1q, the initiator molecule of the complement cascade that increases 300-fold in healthy aging and accumulates with neurodegeneration. We have previously reported that exogenous C1q treatment alters inflammatory gene expression and cell function in human induced pluripotent stem cell-derived microglia (iMG). Here, we test the hypothesis that C1q induced cell changes are modulated by novel C1q receptor, CD44. We first used validated expression of five recently identified C1q receptors at the RNA and protein levels, and then we used proximity ligation assay to validate C1q-receptor binding on the iMG cell surface. CD44 was selected as an initial target and thus CD44 knockout iMG were generated to test whether the C1q response is dependent on CD44. While the C1q-induced inflammatory response was not dependent on CD44, we demonstrate that C1q-CD44 interactions regulate changes in microglial phagocytosis, proliferation, and migration. These data suggest C1q interacts with CD44 on iMG to modulate microglial functions that are critical to health and disease. This data informs future work which will test how C1q-CD44 interactions are altered in neurodegenerative disease and if these interactions could be modulated as a therapeutic target.

## INTRODUCTION

Aging and neurodegenerative disease trigger microglia, the resident immune cells of the central nervous system (CNS), to transition from a homeostatic state into an inflammatory state. This transition leads to neuroinflammation that has either beneficial or detrimental roles depending on the context; however, understanding the mechanisms regulating microglial transition into an inflammatory state will inform therapeutic development for a wide range of neurodegenerative disorders.

One molecular regulator of interest is complement protein C1q, which has been reported to polarize microglial state and drive secretion of inflammatory cytokines in primary rat microglia cultures (Färber et al., 2009). We recently demonstrated that this phenomenon is conserved in human microglia, reporting that exogenous C1q treatment increases inflammatory gene expression and changes in organelle and cell functionality in human induced pluripotent stem cell (hiPSC)-derived microglia (iMG) (Sakthivel et al., 2024). For these studies, we utilized recent advances in robust and reproducible differentiation of iMG protocols, which have unlocked new ways to gain insights into human mechanisms of neuroinflammation. Indeed, in this paradigm, iMG display highly similar transcriptomic signatures to recently isolated primary human microglia, while also maintaining the heterogeneity in subpopulations characteristic of *in vivo* microglia (Abud et al., 2017; Hasselmann et al., 2019; McQuade et al., 2018), identifying a key platform to investigate mechanisms of inflammation in human cells.

In stark contrast to the traditional function of C1q within the autocatalytic complement cascade, these findings highlight the capacity of C1q to influence cellular behavior as a single ligand that is conserved across rodents and humans. In support of the notion that C1q can act independent of the cascade, C1q influences neural stem cell fate and migration (Benavente et al., 2020; Hooshmand et al., 2017), developmental synaptic plasticity (Stevens et al., 2007), and astrocyte activation (Liddelow et al., 2017). While increasing bodies of work suggest a distinct function of C1q in the CNS working as a single ligand, a mechanistic understanding of how C1q drives these complex changes in cellular function is currently lacking. In this regard, we have suggested that C1q can participate in receptor-mediated signaling. Indeed, we recently conducted unbiased screening of plasma membrane proteins via C1q-pulldown and mass spectrometry in human neural stem cells, identifying five novel candidate C1q receptors: ADCY5, BAI1, CD44, cMET, and GPR62 (Benavente et al., 2020). Supporting the notion of C1q-receptor interactions, we showed that C1q binds to the transmembrane glycoprotein CD44 to regulate human neural stem cell migration *in vitro* and *in vivo* after a spinal cord injury (Benavente et al., 2020).

We therefore hypothesized that C1q would similarly behave as a ligand with receptors on the microglial plasma membrane to regulate neuroinflammation and microglial functions. We utilized iMG to validate expression of all five candidate C1q receptors and their interactions with C1q on the microglial cell surface. Moreover, we found that C1q-CD44 interactions modulate C1q-dependent changes in phagocytosis, proliferation, and migration. These data highlight, for the first time, a mechanism underlying how C1q as a single molecule can influence microglial functions, suggesting that these interactions are critical to inflammation and disease.

## RESULTS

### Human microglia express candidate C1q receptors at the RNA and protein levels

**Figure 1:**
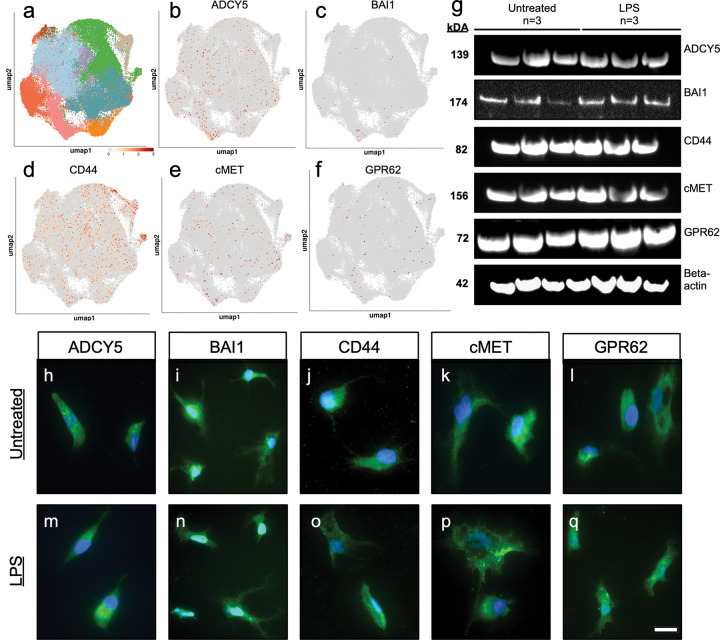
iMG express all five candidate C1q binding partners. Unbiased screening for C1q binding partners in human neural stem cells identified five novel signaling candidates in a previous study. (a-f) Single cell RNA sequencing data of human microglia (a) confirms transcriptomic expression of ADCY5 (b), BAI1 (c), CD44 (d), cMET (e), and GPR62 (f). (g) Western blot additionally validates expression of all five signaling candidates in untreated (n=3, left) and LPS-treated (n=3, right) iMG. (h-q) Immunocytochemistry demonstrates positive expression of each target signaling candidate (green) and Hoechst (blue) in iMG. Untreated and LPS-treated iMG express ADCY5 (h,m), BAI1 (i,n), CD44 (j,o), cMET (k,p), and GPR62 (i,q). Scale bar = 10 μm.

We tested whether the C1q candidate receptors are expressed in human microglia in addition to neural stem cells by evaluating a publicly available integrated single-cell, human microglia RNA sequencing data set that combined 90,716 nuclei/cells across nineteen datasets (Figure 1A). These data validated transcriptomic expression of all five candidate receptors in human microglia (Figure 1B–1F). All candidate C1q receptors showed transcriptional expression, however, expression of CD44 was strikingly higher than other candidate receptors. Receptor expression patterns were not correlated with any of the nine subpopulations identified in this dataset, suggesting a pan-microglial role for C1q-receptor interactions. These data confirm that human microglia express all candidate receptors at the RNA level and that CD44 expression is the highest.

Although ADCY5, BAI1, cMET and GPR62 showed lower expression patterns compared with CD44, we predicted that this could be due to stable protein expression on the cell surface, a particular feature for G protein-coupled receptors like BAI1 and GPR62. We therefore probed receptor expression at the protein level. We evaluated receptor expression dynamics based on activation state in untreated and lipopolysaccharide (LPS)-treated iMG by western blot (Figure 1G) and immunocytochemistry (Figure 1H–Q). LPS is a commonly used stimulus to drive microglial activation *in vitro*, acting via TLR4 to drive increased expression of inflammatory genes. We confirmed stable presence of all candidate receptors at the protein level in untreated and LPS-treated microglia (Figure 1G–1Q), suggesting that activation state of microglia does not modulate receptor expression. Moreover, consistent with the hypothesis that transcriptional analysis may not accurately correlate with protein expression, immunocytochemistry revealed that all microglia displayed equal expression of all receptors at the protein level.Overall, these data validate expression of the candidate C1q receptors at the RNA and protein levels in microglia and suggest that C1q may be acting as a ligand with these receptors, as it does in neural stem cells.

### C1q interacts with five novel C1q receptors on the microglial plasma membrane

**Figure 2:**
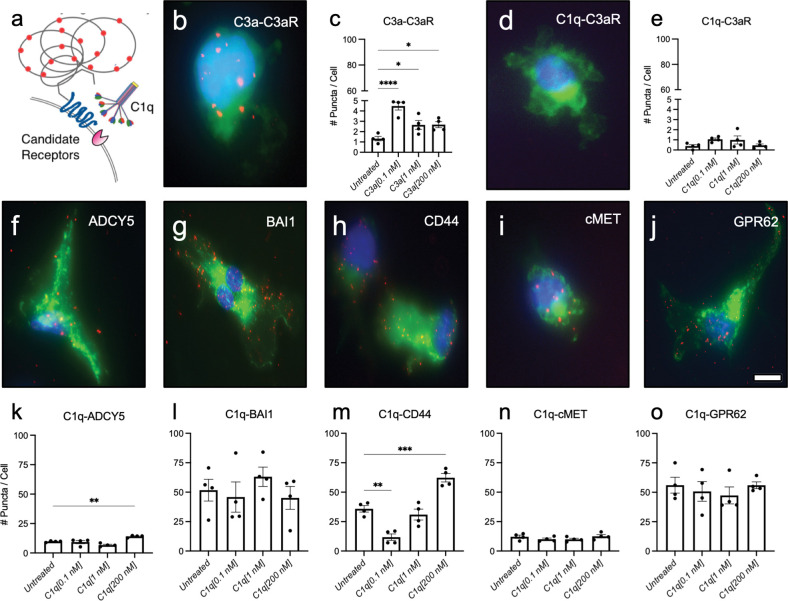
PLA verifies C1q-binding partner interactions at the microglial plasma membrane. (a) PLA shows quantifiable positive red fluorescent puncta when two target proteins are interacting within <30 nm. (b-e) PLA validation shows a representative image of a matched ligand-receptor positive control (C3a-C3aR) in (b-c) and a mismatched ligand-receptor negative control (C1q-C3aR) in (d-e). (f-o) PLA representative images and quantification of C1q with novel signaling candidates C1q-ADCY5 (f,g); C1q-BAI1 (h,i); C1q-CD44 (j,k); C1q-cMET (l,m); C1q-GPR62 (n,o). Membrane staining (green) shown with Alexa 488 conjugated wheat germ agglutinin and nucleus shown with Hoechst (blue). Graphs represent quantification of the average number of fluorescent puncta/cell in 13 random pictures/well. n= 2 replicates (wells) were quantified across two independent experiments; mean ± SEM. Statistical analysis by one-way ANOVA. No significant difference in C1q-C3aR negative control, C1q-cMET, and C1q-GPR62 groups. All other groups showed significance for one-way ANOVA (p≤0.05) and analysis was followed by Dunnett’s multiple comparisons test as indicated. *p≤0.05, **p≤0.01, ***p≤0.001, ****p≤0.0001. Scale bar = 5 μm.

Confirmation of receptor expression does not mean that C1q plays a conserved role as a ligand for these receptors in microglia. Therefore, we next tested whether C1q interacts with these receptors on the plasma membrane by employing proximity ligation assays (PLA), in which positive red fluorescent puncta indicate protein-protein interactions (Figure 2A). iMG were treated with purified C1q for 30 minutes at three physiologically relevant concentrations secreted by neutrophils (C1q[0.1 nM]), macrophages (C1q[1 nM]), or as present in the blood/serum (C1q[200 nM]) as previously described by our lab (Hooshmand et al., 2017). PLA validation was performed with a positive matched ligand control (C3a-C3aR) and a negative mismatched ligand control (C1q-C3aR) (Figure 2B–E). We quantified protein interaction by analyzing the number of fluorescent puncta per cell. Figure 2F–J shows representative images of PLA detection of C1q-ADCY5, C1q-BAI1, C1q-CD44, C1q-cMET, and C1q-GPR62 interactions. Consistent with the fact that microglia are the largest producers of C1q in the CNS (Fonseca et al., 2017), we observed interactions between C1q and each candidate receptor in untreated (baseline) conditions in which no exogenous C1q was added. Interestingly, all receptors showed different levels of baseline interactions with C1q in the untreated condition (Figure 2K–O). This data could suggest either varying levels of each candidate receptor at the plasma membrane or varying affinities between each receptor and C1q. Thus, we confirm that C1q interacts with these five candidate receptors on the microglial cell surface.

**Figure 3:**
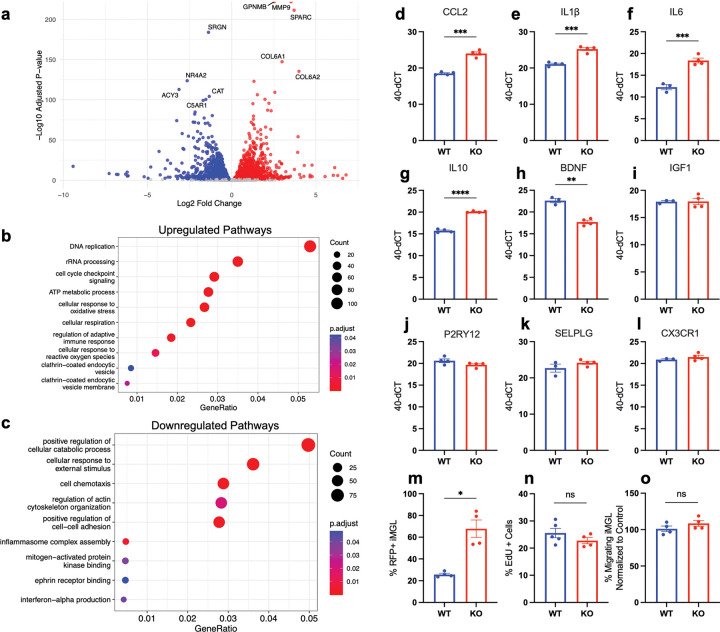
CD44 KO iMG display an altered transcriptomic signature and phagocytic baseline compared to WT iMG. (A) Differential gene expression analysis between WT and CD44 KO iMG revealing significant upregulation (red) and downregulation (blue). (B-C) Gene ontology overrepresentation analysis reveals pathways that are significantly enriched (B) and repressed (C) in CD44 KO iMG compared to WT. (D-I) qPCR analysis of WT vs KO cells reveals changes in representative pro-inflammatory genes (D-F) and anti-inflammatory genes (G-I), but not change in homeostatic genes (J-L). (M) CD44 KO iMG display an increase in RFP, compared to WT iMG, following treatment with pHrodo particles, indicative of increased phagocytosis in KO cells. (N) WT and CD44 KO iMG both exhibit about 20–25% proliferation as quantified by EdU incorporation and flow cytometry. (O) Transwell migration assay reveals that WT and CD44 KO iMG display similar migration. n= 3–5 biological replicates; mean ± SEM. Statistical analysis by Student’s T-test. *p≤0.05, **p≤0.01, ***p≤0.001, ****p≤0.0001.

We evaluated the dynamics of these interactions in response to C1q treatment, which we and others have shown induces microglial activation and polarization (Färber et al., 2009; Sakthivel et al., 2024). We identified that the number of C1q-BAI1(Figure 2L), C1q-cMET (Figure 2N) and C1q-GPR62 (Figure 2O) interactions did not change in response to C1q treatment. These data suggest these interactions modulate a function that is not dependent on cell activation state, or alternatively that these receptors may have a low equilibrium dissociation constant and become saturated with autocrine C1q and/or C1q at low doses. In contrast, C1q-ADCY5 (Figure 2K) and C1q-CD44 (Figure 2M) interactions showed an increase in the number of interactions with C1q in response to treatment. These data suggest these receptors could be involved in a cellular function associated with the microglial state observed following C1q treatment. Validation of C1q interactions with these candidate receptors therefore reveals potential new mechanisms by which C1q may regulate microglia state and function. Critically, of the candidate receptors, CD44 displayed not only the highest level of transcriptomic expression (Figure 2D), but also the most striking changes in number of interactions following C1q treatment (Figure 3M). We therefore selected CD44 for proof of concept analysis to investigate the biological role of C1q-CD44 interactions on microglia.

### CD44 KO iMG display an altered transcriptomic profile in comparison with WT iMG

With the goal to test how the response to C1q is altered without CD44, we generated wildtype (WT) and CD44 knockout (KO) hiPSC using CRISPR/Cas9 gene editing. Guide RNAs generated a 47 bp deletion in exon 2 of CD44 (**Supp. Figure 1A-C)** that led to a premature stop codon and successful generation of a knockout cell line, as validated by absence of CD44 expression in iMG via immunocytochemistry **(Supp. Figure 1D-E)** and western blot **(Supp. Figure 1F).** Of note, CD44 is involved in numerous functions that are critical to microglia, such as neuroinflammation, migration, and cell-cell adhesion. CD44 also has multiple ligands produced by microglia themselves, including not only C1q, but also hyaluronic acid, osteopontin, and fibronectin. Thus, we anticipated that disruption of CD44 at baseline would alter microglial transcriptional signature and function, even in the absence of stimulation with an exogenous ligand. However, we reasoned that these cells could still provide a tool to assess the response to C1q so long as the capacity to respond to extracellular cues is intact. We therefore confirmed that generation of a CD44 KO did not exhibit a level of altered function/polarization that would prevent analysis of responses to C1q by bulk RNA sequencing and functional analyses.

As anticipated, bulk RNA sequencing revealed that CD44 KO iMG showed striking changes in RNA expression when compared to WT iMG, with 2,225 significantly upregulated genes and 2,110 significantly downregulated genes (Figure 3A). Gene ontology by overrepresentation analysis revealed that CD44 KO iMG significantly upregulated genes related to DNA replication and cell cycle checkpoint signaling, consistent with the role of CD44 isoforms in numerous cancer types (Figure 3B). CD44 KO iMG also exhibited upregulation of numerous pathways associated with pro-inflammatory or disease states, such as cellular response to oxidative stress, regulation of adaptive immune response, and cellular response to reactive oxygen species (Figure 3B). We also observed an increase in clathrin-coated endocytic vesicle components, in accordance with the primary function of microglia as phagocytic cells (Figure 3B). Increased expression of these pathways with no stimulus present implies that CD44 KO iMG are polarized towards an inflammatory or disease state at baseline.

CD44 KO iMG repressed pathways related to cellular responses to external stimuli and signaling pathways, as expected due to the genetic deletion of a receptor (Figure 3C). CD44 KO iMG also decreased expression of cellular catabolic process, suggesting CD44 plays a role in autophagy, macromolecule turnover, or regulating energy production (Figure 3C). We moreover observed that CD44 KO iMG downregulate cytoskeleton organization and cell-cell adhesion pathways (Figure 3C); again, this is consistent with the established role of CD44 in regulating migration and the extracellular matrix. Lastly, we found that CD44 KO iMG repressed inflammasome, MAPK, and interferon pathways (Figure 3C). In support of the bulk RNA sequencing data, qPCR validated increased pro-inflammatory gene expression (CCL2, IL1β, IL6) in CD44 KO iMG versus WT iMG (Figure 3D–F). CD44 KO iMG also upregulated IL-10 expression (Figure 3G), and downregulated IGF1 expression (Figure 3H), consistent with an inflammatory state. Conversely, CD44 KO showed no change in expression of anti-inflammatory marker BDNF (Figure 3I), or homeostatic markers P2RY12, SELPLG, and CX3CR1 (Figure 3J–L). Overall, these data reveal that CD44 KO alters a number of inflammatory signaling pathways in iMG.

We considered the possibility that these transcriptomic changes are due to disruption of CD44 interactions with autocrine C1q that is produced by microglia themselves. To test this hypothesis, WT iMG were treated with a C1q neutralizing antibody (NAb) for 24 hours to block C1q function at baseline before bulk RNA sequencing. Our lab has extensively characterized this NAb in blocking C1q function, as previously published (Hooshmand et al., 2017). In stark contrast to the effect of CD44 KO, we found that blocking autocrine C1q had a minor influence on microglial transcriptional state, altering the expression of fewer than 100 genes **(Supp. Figure 2A)**. Therefore, it is unlikely that disruption of C1q-CD44 interactions is the predominant driver for the altered polarization state observed in CD44 KO iMG.

We also evaluated whether the transcriptional changes in phagocytosis, proliferation, and migration identified in CD44 KO iMG were associated with baseline changes in function. WT and CD44 KO iMG phagocytosis was quantified by flow cytometry following a one hour incubation with pHrodo particles, which become fluorescent upon internalization into an endosome and are indicative of phagocytosis. CD44 KO iMG displayed increased phagocytosis rates when compared to WT iMG (Figure 3M), consistent with the interpretation that disruption of CD44 drives a more inflammatory microglial activation state. In contrast, CD44 KO iMG did not exhibit changes in proliferation via EdU incorporation and flow cytometry, or changes in migration via transwell migration assay (Figure 3N–O). These data show that, despite striking transcriptional changes and alterations in phagocytosis, CD44 KO iMG retain normal proliferation and migration profiles.

**Figure 4:**
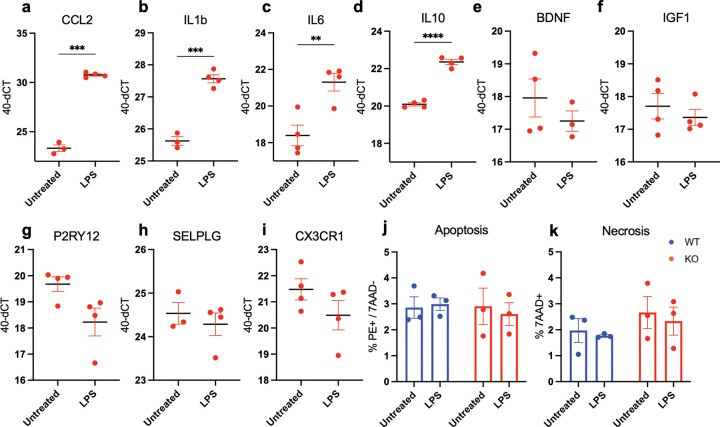
CD44 KO iMG retain the capacity to respond to LPS and do not show changes in cell death. (A-I) iMG remained untreated or were treated with 100 ng/mLLPS. Cells were lysed following a 24 hour treatment and RNA was collected to observe changes in representative inflammatory (A-C), anti-inflammatory (D-F), and homeostatic genes (G-I). LPS treatment drives an increase in CCL2, IL1β, IL6, and IL10 expression in CD44 KO iMG, suggesting there is no ceiling effect. n= 3–4 biological replicates; mean ± SEM. Statistical analysis by Student’s T-test. **p≤0.01, ***p≤0.001, ****p≤0.0001. (J-K) Neither WT or CD44 KO iMG do not show changes in apoptosis (J) or necrosis (K) at baseline or in response to LPS as shown by Annexin/7AAD expression quantified by flow cytometry. n=3 biological replicates; mean ± SEM. Statistical analysis using two-way ANOVA (p≤0.05), followed by Sidak’s multiple comparisons test as indicated. *p≤0.05, **p≤0.01, ***p≤0.001, ****p≤0.0001.

Finally, we tested if CD44 KO microglia retained the capacity to respond to external stimuli, which is essential to enable study of C1q responses in these cells. Like WT iMG **(Supp. Figure 3)**, CD44 KO iMG retained the capacity to respond to LPS, as quantified by increased CCL2, IL1β, IL6, and IL10 expression via qPCR (Figure 4A–I). This response demonstrates that CD44 KO did not elicit a ceiling effect in activation state that would preclude the ability to detect further inflammatory modulation. Moreover, CD44 KO iMG do not show altered apoptosis or necrosis levels at baseline or in response to LPS (Figure 4J–K), highlighting that disruption of CD44 does not lead to premature cell death. These data demonstrate that, despite an altered baseline state, CD44 KO iMG can respond to extracellular cues and thus can be used to study the microglial response to C1q. We therefore next investigated how C1q influences microglial inflammation and whether this is dependent on CD44.

### C1q modulation of the inflammatory transcriptional response is mediated in part through CD44

We used bulk RNA sequencing to evaluate microglial transcriptional state in response to treatment with exogenous C1q. WT iMG were treated with C1q[200 nM] for 24 hours, resulting in a significant upregulation of 89 genes and downregulation of 65 genes (Figure 4A). Gene ontology analysis revealed that C1q treatment enriched pathways related to cytokine production and immune/inflammatory response in WT iMG. C1q also led to the upregulation of genes associated with EGF, ERBB, NF-κB, and TLR2 signaling pathways, all of which are associated with pro-inflammatory microglia (Figure 4B). Conversely, C1q repressed pathways related to extracellular matrix and cellular lumen in WT iMG (Figure 4C). These data are consistent with the notion that C1q polarizes microglia to a pro-inflammatory phenotype by upregulating cytokine expression and pro-inflammatory signaling pathways.

We next evaluated the effect of C1q treatment on microglial transcriptional state in CD44 KO iMG. CD44 KO iMG were treated with C1q[200 nM] for 24 hours, resulting in significant upregulation of 94 genes and downregulation of 72 genes (Figure 4D). Of the differentially expressed genes, 13 and 6 genes, respectively, were conserved from the WT iMG response, suggesting the transcriptional effect of C1q is distinct between WT and CD44 KO iMG. These data identify unique transcriptional programs between WT and KO iMG, however, the pro-inflammatory response to C1q remained largely intact at the pathway level. Indeed, C1q triggered a dramatic enrichment of positive regulation of cytokine production and NF-κB signaling in CD44 KO iMG, consistent with that observed in WT iMG (Figure 4E). We validated this by qPCR, confirming that C1q treatment increased CCL2 and IL1β expression in CD44 KO iMG, as in WT iMG (**Supp. Figure 4).** Similarly, C1q treatment repressed pathways related to vesicle lumen, indicative of changes in organelle organization or secretory pathways, in both WT and CD44 KO iMG (Figure 4F). These data highlight aspects of the C1q transcriptional response that are not dependent on CD44.

To probe what aspects of the C1q transcriptional response are dependent on CD44, we analyzed the interaction effect between genotype and treatment, extracting genes that exhibited the least versus most change between WT and CD44 KO iMG in response to C1q treatment as identified by log_2_ fold change. C1q similarly regulated many genes in WT and CD44 KO iMG (Figure 4G), however, we identified 25 genes that exhibited a significant difference in expression when comparing the response of WT and CD44 KO iMG to C1q treatment (Figure 4H). These were accompanied by identified differences at the pathway level by gene ontology. In particular, CD44 KO iMG exhibited a unique upregulation of type I interferon production and protein kinase C binding in response to C1q (Figure 4E). Together, these data highlight that CD44 modulates the inflammatory response to C1q, as seen by both conserved (Figure 4G) and unique (Figure 4H) **gene expression patterns between WT and KO**, identifying that C1q induces a pro-inflammatory transcriptional program that is partially modulated through CD44.

### CD44 regulates C1q-induced changes in phagocytosis, proliferation, and migration

Lastly, we tested the hypothesis that C1q-CD44 interactions modulate microglia at the functional level. We and others have previously shown that C1q treatment leads to increased microglial phagocytosis, decreased proliferation, and increased migration (Sakthivel et al., 2024). Moreover, the bulk RNA sequencing data generated in this study highlight differential expression of genes and pathways related to phagocytosis, proliferation and migration. One possibility is that C1q treatment upregulates the expression of other CD44 ligands, indirectly regulating the functional cellular behavior. To test this hypothesis, we investigated the effect of C1q treatment on these ligands in RNAseq. No changes in alternative CD44 ligand expression were identified **(Supp. Figure 5A)**. Having excluded this mechanism, we then sought to probe the functional consequences of direct C1q-CD44 interactions by testing the effect of C1q on microglial function using WT versus CD44 KO iMG.

**Figure 5:**
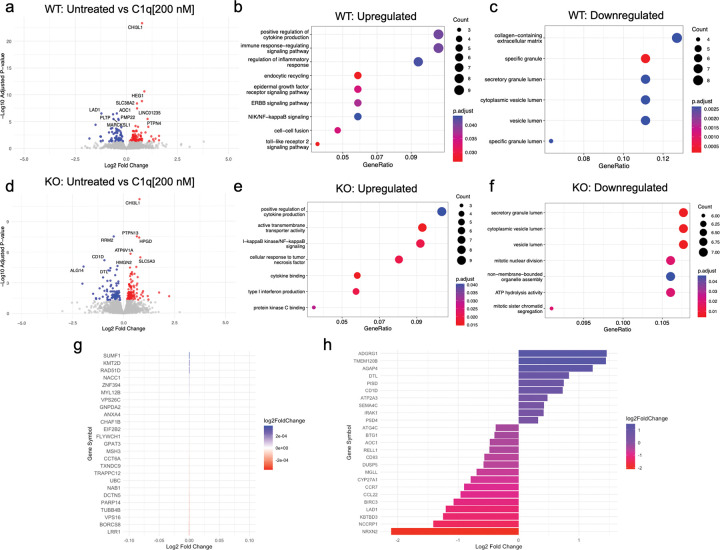
CD44 KO iMG do not transcriptionally respond to C1q as WT iMG do. (A-C) Differential gene expression analysis of WT iMG untreated versus C1q[200 nM] treatment for 24 hours (A) and overrepresentation analysis highlighting enriched (B) and repressed (C) pathways. (D-F) Differential gene expression analysis of CD44 KO iMG untreated versus C1q[200 nM] treatment for 24 hours (D) and overrepresentation analysis highlighting enriched (E) and repressed (F) pathways. (G-H) Barplots highlighting the top 25 most differentially expressed genes (G) and least differentially expressed genes (H) when comparing the response to C1q in WT versus CD44 KO iMG.

We first assessed phagocytosis because of the established function of C1q as an opsonization molecule. As expected, C1q triggered an increase in phagocytosis rate in WT iMG as quantified by flow cytometry after C1q and phRodo incubation. In contrast, C1q did not alter phagocytosis in CD44 KO iMG (Figure 5A), suggesting that CD44 is required for microglia to exhibit C1q-induced phagocytosis. We additionally tested changes in cell proliferation by EdU incorporation and flow cytometry. C1q[200 nM] decreased proliferation in WT iMG, and this effect was dependent on CD44 expression, as it was lost in CD44 KO iMG (Figure 5B).

We also tested the effect of LPS on microglial proliferation in WT and CD44 KO iMG. LPS is known to interact with TLR4 to activate an immune response (Lu et al., 2008). Interestingly, TLR4 can act as a co-receptor complex with CD44 to modulate stem cell proliferation (Riehl et al., 2015), suggesting that CD44 could serve as an integration point for proliferation signals from both pathways in microglia. Consistent with previous reports in primary rat microglia (Vay et al., 2018), we found that LPS treatment decreased WT iMG proliferation in our model, and that this decrease was also dependent on CD44 expression, as the effect was lost in CD44 KO iMG (Figure 5B).

One possibility is that LPS alters microglial proliferation via CD44-TLR acting in a co-receptor complex.Alternatively, this LPS-induced change in proliferation could be due to increased C1q production **(Supp. Figure 5B)** and a downstream increase in autocrine C1q-CD44 interactions. We therefore tested this latter possibility by treating iMG with a C1q NAb to investigate whether LPS alters microglial proliferation via autocrine production of microglial C1q. While C1q blockade via NAb at baseline decreased WT iMG proliferation rates, LPS and NAb treatment together did not reverse the LPS-induced decrease in proliferation **(Supp. Figure 5C)**. Neither LPS nor NAb treatment drove changes in apoptosis or necrosis **(Supp. Figure 5D-E)**, suggesting this is a true proliferation phenotype. Thus, autocrine C1q is required for baseline maintenance of microglial proliferation, but LPS-induced decreases in proliferation are not dependent on autocrine C1q. These data highlight the novel possibility that, in human microglia, CD44 serves as an integration point for both C1q and LPS-TLR4 signals in modulating microglial proliferation.

We additionally probed a role of CD44 in modulating C1q-mediated chemotaxis. We and others have previously shown that microglia migrate towards C1q (Lefebvre, 2014; Sakthivel et al., 2024). Accordingly, we tested if this effect is dependent on CD44 using a transwell migration assay. We found that CD44 KO iMG did not migrate towards C1q, while WT iMG did (Figure 5C). In contrast, both CD44 KO and WT iMG exhibited migration towards ADP as a positive control (Figure 5C), suggesting CD44 KO iMG retained the capacity to migrate in response to external cues. Therefore, these data demonstrate that C1q-CD44 interaction is a novel mechanism for microglial chemotaxis. Critically, C1q did not alter apoptosis (Figure 5D) or necrosis (Figure 5E) in either WT or CD44 KO iMG. In sum, these data demonstrate that C1q-CD44 interactions are essential for modulation of microglial phagocytosis, proliferation, and migration, revealing a novel mechanism by which microglial function is regulated.

## DISCUSSION

While CD44 has several ligands that have been well-studied (i.e. osteopontin, hyaluronic acid), here we investigate the relationship between CD44 and its most recently identified ligand, complement protein C1q (Benavente et al., 2020). In this study, we hypothesized that C1q signals through CD44 to modulate microglial behavior via receptor-mediated signaling. We first confirmed transcriptomic/proteomic expression of recently identified C1q receptors in iMG, and then validated C1q-candidate receptor interactions using PLA. CD44 was identified as an initial target based on its high expression patterns and the striking increase in C1q-CD44 interactions following C1q treatment. Overall, we demonstrate that CD44 is required for microglia to exhibit C1q-induced changes in phagocytosis, proliferation, and migration. These data suggest that C1q-CD44 interactions modulate functions that are critical for microglia, and that these interactions could be important for both homeostasis and disease.

Microglia are constantly surveying their environment and transition into disease-associated microglia (DAM) based on microenvironmental signals. DAM rapidly proliferate, migrate towards regions of disease, phagocytose debris and pathogens, and secrete inflammatory cytokines to facilitate neuroinflammation. Notably, one DAM marker that has been identified across numerous disease models is CD44. Microglial CD44 expression is elevated following stroke (Sawada et al., 2020), ALS (Sawada et al., 2020), glioma (Du et al., 2022), and Alzheimer’s disease (Rangaraju et al., 2018). CD44+ microglia/macrophages appear in the infarct region following ischemia and CD44 expression persists into the chronic phase post-stroke (Sawada et al., 2020). Both astrocytes and microglia display increased CD44 expression at disease onset that continuously increases in rodent models of ALS (Matsumoto et al., 2012). Microglia/macrophages within the glioma microenvironment also express high levels of CD44 correlated with tumor progression in humans (Xiao et al., 2022). Similarly, expression of CD44 is significantly elevated in post-mortem Alzheimer’s disease brain when compared to age-matched control tissue (Pinner et al., 2017). Moreover, C1q expression dramatically increases 300-fold across healthy aging and further accumulates in neurotrauma and neurodegeneration (Stephan et al., 2013). This literature demonstrates the abundance of both C1q and CD44 in the disease microenvironment and highlights the potential for C1q-CD44 interactions to modulate DAM state via a conserved mechanism across neurodegenerative disease.

Numerous studies have found anti-CD44 antibody treatment inhibits inflammation in rodent models of arthritis(Zeidler et al., 1995), cutaneous inflammation(Camp et al., 1993), multiple sclerosis(Brocke et al., 1999), vascular leak syndrome(Rafi-Janajreh et al., 1999), and Parkinson’s disease (Wang et al., 2022). Moreover, blocking CD44 inhibits LPS-induced TLR4 expression and NF-κB p65 nuclear translocation in BV2 murine microglial cells (Wang et al., 2019). Conversely, CD44 stimulation via monoclonal antibodies or soluble hyaluronan triggers the release of inflammatory cytokines TNF-α and IL-1 in both human monocytes and murine macrophages (Webb et al., 1990; Noble et al., 1993). Thus, CD44 has a very well-established role in regulating inflammation in the CNS.

Based on this data, we hypothesized that CD44 would similarly be required for C1q-induced neuroinflammation. In contrast, however, we found that CD44 KO iMG retain the ability to upregulate immune response genes following C1q treatment. Several possibilities may provide insight into this result. First, in contrast with our human microglia model, previous CD44 studies have primarily utilized rodent models or immortalized human cell lines. Indeed, human versus mouse microglia show varying expression of neurodegenerative ‘risk genes’, including complement proteins C1q and C3a, highlighting the importance of using human iMG to investigate cellular mechanisms as employed here (Burns et al., 2015; Geirsdottir et al., 2019). Second, previous studies have utilized antibodies or hyaluronan as stimuli, which may induce a distinct mechanistic response when compared to stimulation via C1q. In support of this notion, low versus high molecular weights of hyaluronan differentially interact with CD44 to drive changes in macrophage and endothelial cell function (Karam et al., 2023; Rayahin et al., 2015). In addition to these points, we note that disruption of CD44 by generation of a KO iMG altered the baseline transcriptional program. It is likely that this is due to disruption of cell-cell interactions and cell-extracellular matrix interactions, as CD44 is well known to act as an adhesion molecule (Goodison et al., 1999). Interestingly, while both WT iMG and CD44 KO iMG upregulate similar functional pathways in response to C1q, there is little overlap between the significant differentially expressed genes in WT versus KO iMG, suggesting that CD44 KO iMG exhibit conserved inflammatory responses but via distinct gene expression profiles.

While the transcriptional influence of C1q was not directly mediated by CD44, we did identify that C1q-CD44 interactions modulate functional changes that are critical to microglia, including phagocytosis, proliferation, and migration. While our data are consistent with the additional known roles for CD44, we identify for the first time a mechanistic understanding for how C1q modulates these functions in human microglia. Indeed, CD44 is known to act as a phagocytic receptor in erythrocytes (Vachon et al., 2006) and macrophages (Vachon et al., 2007). Consistent with the conventional role of C1q in opsonization for phagocytosis, our data suggest that C1q interacts with CD44 in microglia to facilitate debris and pathogen clearance. CD44 is also known to modulate proliferation of numerous cell types, including breast cancer cells (Nam et al., 2015), lung cancer cells (Hu et al., 2018), and epithelial cells (Abbasi et al., 1993). We identify, for the first time, a function for CD44 in modulating proliferation in microglia in response to both C1q and LPS, and that this proliferation effect is not dependent on autocrine C1q production. Moreover, a role for CD44 in modulating cell migration has been well-established (Fernández-Tabanera et al., 2023; Nam et al., 2015; Wolf et al., 2020) and we and others have shown that C1q serves as a chemotactic cue for microglia (Lefebvre, 2014; Sakthivel et al., 2024). However, there was no mechanistic understanding for this effect. Here, we show for the first time that iMG migrate towards C1q in a CD44-dependent manner. These data together demonstrate C1q interacts with CD44 to modulate three critical microglial functions that are essential for health and disease.

It is important to note that CD44 has numerous ligands, many of which are produced by microglia themselves, potentially acting in an autocrine manner to self-regulate function. However, transcriptomic expression of CD44 ligands was unchanged in WT and KO iMG at baseline or in response to C1q. Further, we show that autocrine C1q does not regulate iMG baseline transcriptional state or LPS-induced changes in proliferation. We do interestingly identify that autocrine C1q is required for baseline iMG turnover, and that this function is CD44 independent, suggesting that a different interaction partner mediates this effect. Accordingly, future studies will investigate the four other C1q receptors whose expression profiles and interactions with C1q were validated in this manuscript.

Future work will interrogate the downstream cellular pathways underlying CD44 dependent phenotypes. Indeed, CD44 has multiple known mechanisms of action, including regulating signaling cascades, cleavage of its extracellular and intracellular domains, and complexing with other cell surface receptors. Critically, the present study identifies a novel role for C1q-CD44 interactions in regulating microglial function. Interestingly, recent work suggests influences on microglial state and function by both genetics and sex (Kang et al., 2024; Li et al., 2023; Thion et al., 2018; Villa et al., 2018). Expansion of this work to interrogate male and female patient lines, as well as the contributions of genetic factors in disease, will yield new mechanistic insights into how C1q-CD44 interactions modulate microglial function. In sum, we identified a novel mechanism by which C1q directly modulates microglial behaviors through a recently identified receptor, CD44. These data provide the foundation to test if and how these mechanisms are altered in neurodegenerative disease, where both C1q and CD44 expression is elevated.

## MATERIALS & METHODS

### hiPSC Acquisition and Maintenance

University of California, Irvine (UCI) Alzheimer’s Disease Research Center ADRC76 cell line was obtained from human fibroblast-derived hiPSC with informed consent. UCI Institutional Review Board approved the reprogramming and differentiation, and UCI Human Stem Cell Research Oversight Committee approved the hiPSC use and differentiation towards microglia (hSCRO protocol # 3682). hiPSC were grown on 6-well plates that were pre-coated with Matrigel (1 mg/mL; BD Biosciences 356231). Cells were maintained in a humidified incubator and received daily media changes with mTeSR Plus (Stem Cell Technologies 100–0276). During passaging, media was supplemented with 0.5 μM Thiazovivin (Stem Cell Technologies, 72252). hiPSC were tested for mycoplasma every three months and confirmed to be negative.

### iMG Generation

hiPSC were generated according to the previously published protocol (McQuade et al., 2018). Briefly, hematopoietic progenitors were made using STEMdiff Hematopoietic Kit (Stem Cell Technologies, 5310) and non-adherent CD43 + hematopoietic progenitors were collected at the end of the differentiation. Then, these cells were plated into iMG complete medium (DMEM/F12, 2X insulin-transferrin-selenite, 2X B27, 0.5X N2, 1X glutamax, 1X non-essential amino acids, 400 mM monothioglycerol, and 5 mg/mL human insulin freshly supplemented with 100ng/mL IL-34, 50ng/mL TGFb1, and 25 ng/mL M-CSF (Peprotech)) and fed every other day for 24 days. On day 25 and day 27, cells were fed iMG maturation medium (iMG complete medium, 100 ng/mL CD200 (Novoprotein), 100 ng/mL CX3CL1 (Peprotech); cells were ready for experimentation on day 28. Cells were incubated with purified C1q (MyBioSource, MBS147305) as indicated.

### Evaluation of public scRNA-seq data set

The publicly available scRNA-seq dataset from (Martins-Ferreira et al., 2025) used to visualize the expression of novel C1q receptors in human microglia.

### Western Blot

Cell pellets were washed once with ice-cold PBS and lysed with RIPA buffer (Thermo, 89900) containing protease (Roche, 5892970001) and phosphatase inhibitors (Roche, 4906845001) for 30 minutes on ice with gentle agitation. Cell lysates were then centrifuged at full speed for 10 minutes at 4°C, and supernatants containing isolated protein were collected into fresh tubes. Protein concentration was determined by BCA and equal amounts of total proteins were resolved in SDS-PAGE precast 4–12% gradient gels (Invitrogen, NP0321BOX) and transferred to PVDF membranes preactivated with methanol and equilibration buffer (GenScript, eBlot L1). PVDF membranes were reactivated with methanol and equilibration buffer following transfer, then blocked with BSA/TBST solution at 4°C for 30 minutes. Receptors were probed by immunoblotting with primary antibody as indicated in Table 1 at 4°C overnight orbital shaking. Following three TBST wash steps, bound antibodies were detected using species-specific HRP-conjugated donkey serum secondary antibody (Thermo, A16029). Secondary antibodies were incubated (1:5000) at room temperature for 1 hour. Immunoblotting results were visualized using chemiluminescent ECL western blot substrate (Thermo, 34075).

### Immunocytochemistry

iMG were fixed in 4% paraformaldehyde for 15 minutes. Cells were blocked with PBS and 5% donkey serum for 10 minutes at room temperature. iMG were stained with primary antibodies as indicated in Table 1 and incubated for 2 hours at room temperature. Cells were then washed with PBS 3 times for 5 minutes each. iMG were stained with secondary primary antibodies (Thermo Fisher, 711–606-152, 1:5000)and nuclear counterstain Hoescht (1:500) for 1 hour at room temperature in the dark. iMG were then washed with PBS 3 times for 5 minutes each before cover slipping with fluoromount mounting solution.

### Proximity Ligation Assay

To validate cell surface C1q-receptor interactions, mature iMG were collected and plated on eight-well glass chamber slides in complete media (50,000 cells/well). Cells were incubated with purified C1q (MyBioSource, MBS147305) at concentrations of [0.1 nM], [1 nM], or [200 nM] for 30 minutes (full receptors) or 3 hours (CD44 ICD) at 37°C. Cells were then fixed with 4% PFA for 15 minutes at room temperature. For investigation of intracellular C1q-receptor interactions only, iMG were permeabilized using 3% Triton X-100 solution for 10 minutes at room temperature and followed by 3 PBS washes. Following fixation and/or permeabilization, cell cytoplasm was stained for 10 minutes at room temperature with wheat germ agglutinin (WGA, Alexa Conjugate 488, Invitrogen, W11261, 1:1000). PLA was then performed using the Duolink In Situ Red Starter Kit Mouse/Rabbit (Sigma-Aldrich, DUO92101) following the manufacturer protocol. This began by blocking the cells for 30 minutes at 37°C using Duolink Blocking Solution. Primary antibodies indicated in Table 1 were incubated for 2 hours at 37°C and washed with Duolink in situ wash buffer A. Cells were then incubated with species-specific secondary antibody-complimentary PLA probes (1:5) for 1 hour in humidified chambers at 37°C. PLA probes were ligated for 30 minutes at 37°C, then amplified for 100 minutes at 37°C. Following amplification, the slides were washed with Duolink in situ wash buffer B and then cover-slipped using Duolink in situ DAPI mounting media (Sigma-Aldrich, DUO82040). For analysis, 12 random images per well were captured at 40x using 0.275 μm intervals with a Zeiss microscope system. The number of red positive punctae was assessed manually using MBF Bioscience StereoInvestigator, providing an average fluorescent puncta count per cell. All experiments were performed in biological and technical duplicates.

### CD44 KO hiPSC Generation

Ribonucleoprotein (RNP) delivery vectors were generated by first annealing Cas9 tracrRNA (IDTDNA, 1072533) and two customized gRNAs targeting exon 2 of CD44 at 95°C for 10 minutes. Guide RNA 1 was 5’ AATATAACCTGCCGCTTTGC 3’ and guide RNA 2 was 5’ TCGCTACAGCATCTCTCGGA 3’. The annealed gRNA was slowly cooled then complexed with HiFiCas9 enzyme (IDTDNA, 10810620) at a 1.2:1 molar ratio for 15 minutes at RT. hiPSC were dissociated by Accutase incubation for 1 minute at 37°C and resuspended in 65 μL nucleofection buffer (Lonza, VPH-5022) with Cas9 RNP and electroporation enhancer (STEMCELL Technologies) prior to nucleofection (Lonza, Amaxa Nucleofector program B-016). Transfected cells were plated in complete media containing mTeSR Plus (STEMCELL Technologies), 0.2 μm Thiazovivin (STEMCELL Technologies) and CloneR (STEMCELL Technologies) to recover for 24 hours. Cells were then plated into as single cells for clonal selection by visual screening daily. DNA was isolated (Qiagen, 69504) and the edit was validated by Sanger sequencing. Clones that passed screening were differentiated into microglia and knockout was again validated by immunocytochemistry and western blot.

### RNA Isolation and RT-qPCR

RNA isolation and RT-qPCR were conducted as described in Sakthivel et al. 2024. Cells were harvested and lysed 24 hours post treatment utilizing the Qiagen RNeasy micro extraction kit (Qiagen, 74004). Initial lysis and protein denaturation were conducted using the RLT master mix with 1% β-mercaptoethanol followed by DNAse I treatment to degrade residual genomic DNA. Purified RNA were then reverse-transcribed into complementary DNA (cDNA) using commercially available high-Capacity RNA-to-cDNA kit (Applied Biosystems, 4387400). Integrity and purity of RNA samples were verified using a nanodrop spectrophotometer. RT-qPCR reactions were performed in 384 well qPCR plates at 10μL volumes using curated panel of primers listed in Table 2. Reaction master mix consisted of 2X TaqMan Gene Expression Fast Master Mix (Applied Biosystems, 4444557), 20X TaqMan Gene Expression primers, template cDNA, and molecular grade RNase free water. PCR plate was sealed and centrifuged prior to loading into Quantstudio7 Flex. Relative gene expression was determined utilizing the comparative ΔΔCt method (Schmittgen & Livak, 2008). All experiments were performed with four biological replicates and three technical replicates during readouts.

### Bulk RNA Sequencing

RNA integrity (RIN) values and concentrations were determined using the Agilent Bioanalyzer 2100 series and Qubit respectively. Clontech SMART-seq V4 Ultra Low Input kit (Takara Bio) was used for library construction via poly-A enrichment to enrich selection of mRNAs. Quality of DNA libraries and concentrations were determined using high sensitivity DNA assay on the aforementioned Bioanalyzer and Qubit. DNA libraries were quantified using Kapa PCR, normalized to 2mM and then multiplexed for sequencing. Illumina HiSeq 4000 platform was used for sequencing with single read 100 base chemistry. Quality control was performed using FASTQC (Andrews, 2014) to validate the quality of sequencing files prior to alignment. Reads were then pseudoaligned to the human genome using Salmon, and gene level summary of transcripts were obtained via tximport (Soneson et al., 2015). Genes with at least 10 read counts in ≥ 3 samples were retained for differential expression analysis using DESeq2 (Love et al., 2014) and an adjusted p-value (Benjamini–Hochberg) cutoff of 0.05.Volcano plots were generated using the “ggplot” function. Gene Ontology (GO) enrichment analysis was conducted using clusterProfiler (Yu et al., 2012). All bulk RNA-seq analysis was performed in R programming language 4.2.1.

### Flow Cytometry a

Phagocytosis: Microglial phagocytic activity was measured through pHrodo ingestion, which exhibits a red fluorescence in response to acidity. iMG were simultaneously treated with C1q and 1:1000 pHrodo (Thermo Fisher, A10010) and incubated for 1 hour at 37°C. Cells were collected and washed twice with PBS/1% FBS before resuspension in 200 μL PBS/1% FBS prior to acquisition. Proliferation: Cell proliferation was assessed using flow cytometric detection of EdU incorporation. iMG were incubated with EdU (Cayman, 20518) 1:1000 for 24 hours in combination with treatment in the incubator. iMG were collected and fixed with 4% PFA for 15 min prior to detection with the Click- iT^™^ Plus Alexa Fluor^™^ 488 Picolyl Azide Toolkit (Invitrogen, C10641) according to the manufacturer’s instructions. Apoptosis/Necrosis: Apoptosis and necrosis were assessed using PE Annexin V Apoptosis Detection Kit with 7- AAD (Biolegend, 640934) according to the manufacturer’s instructions. All samples were acquired on the BD Fortessa and data were analyzed using FloJo. All experiments were conducted in biological triplicate and technical triplicate.

### Migration Assay

Migration assay was performed using migration assay kit (Sigma, ECM512) as previously described (Sakthivel et al., 2024). Briefly, cells were plated as 4 independent treatment replicates in a 96 well plate with transwells containing 0.5μm pores. Cells were lysed 4 hour incubation at 37°C, labeled with fluorophore, and transferred to a black clear bottom 96 well plate for subsequent plate readout. All experiments were performed in biological and technical replicates

### Statistical Analysis

All experiments were performed with 2–4 biological replicates and 3–4 technical replicates depending on the assay. Gene expression analysis via qPCR was tested for statistical significance with either a one-way ANOVA with a post-hoc Dunnett’s multiple comparisons test or a Student’s t-test. Migration, proliferation, and phagocytosis was tested for statistical significance with either a two-way ANOVA with a post-hoc Sidak’s multiple comparisons test or a Student’s t-test. Proximity ligation assay quantification was tested for statistical significance using a one-way ANOVA with a post-hoc Tukey’s multiple comparisons test.

## Supplementary Material

Supplementary Files

This is a list of supplementary files associated with this preprint. Click to download.

• SuppFigure1.jpg

• SuppFigure2.jpg

• SuppFigure3.jpg

• SuppFigure4.jpg

• SuppFigure5.jpg

## Figures and Tables

**Figure 6:**
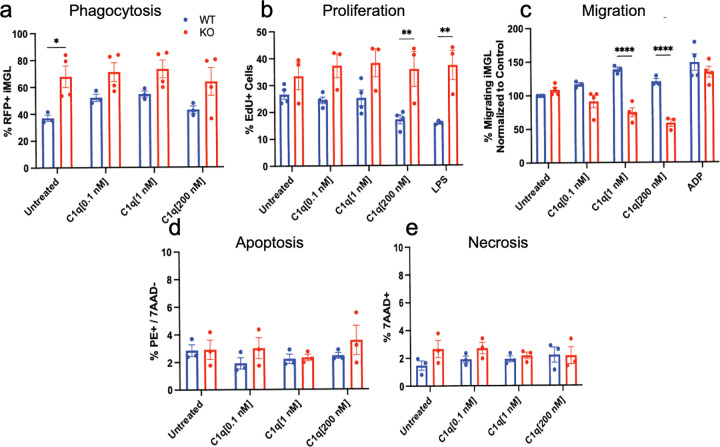
CD44 KO iMG do not functionally respond to C1q as WT iMG do. (A) C1q and pHrodo particle treatment, followed by quantification by flow cytometry, reveals that CD44 KO iMG do not increase phagocytosis in response to C1q as WT iMG do. (B) C1q and EdU treatment, followed by quantification by flow cytometry, reveals CD44 KO iMG do not change proliferation in response to C1q at WT iMG do. (C) Transwell migration assay reveals that CD44 KO iMG do not migrate towards C1q as WT iMG do. (D-E) Neither WT or CD44 KO iMG do not show changes in apoptosis (D) or necrosis (E) at baseline or in response to C1q as shown by Annexin/7AAD expression quantified by flow cytometry. n=3–4 biological replicates; mean ± SEM. Statistical analysis using two-way ANOVA (p≤0.05), followed by Sidak’s multiple comparisons test as indicated. *p≤0.05, **p≤0.01, ***p≤0.001, ****p≤0.0001.

**Table 1:** Antibody applications and dilutions.

Antibody	Species	Manufacturer,catalog #	Application
ADCY5	Rabbit	Abcam, ab66037	ICC: 1:100, PLA: 1:500, WB: 1:500
BAI-1	Rabbit	Abcam, ab135907	ICC: 1:1000, PLA: 1:500, WB: 1:1000
CD44	Rabbit	Abcam, ab51037	ICC: 1:1000, PLA: 1:500, WB: 1:1000
cMET	Rabbit	Abcam, ab5106	ICC: 1:500, PLA: 1:500, WB: 1:1000
GPR62	Rabbit	ThermoFisher, PA3048	ICC: 1:500, PLA: 1:500, WB: 1:1000
C1q	Mouse	Abcam, ab71940	PLA: 1:100
Beta actin	Rabbit	Abcam, ab8227	WB: 1:1000

**Table 2:** Primer assay IDs used for qPCR.

Gene	Assay ID
18S	Hs03003631_g1
CCL2	Hs00234140_m1
IL1β	Hs01555410_m1
IL6	Hs00174131_m1
IL-10	Hs00961622_m1
BDNF	Hs02718934_s1
IGF1	Hs01547656_m1
P2RY12	Hs01881698_s1
SELPLG	Hs05033974_s1
CX3CR1	Hs01922583_s1

## Data Availability

The data that support the findings of this study are available from the corresponding authors upon reasonable request.

## Abbreviations

CNS: Central nervous system
hiPSC: Human induced pluripotent stem cell
iMG: Induced pluripotent stem cell-derived microglia
LPS: Lipopolysaccharide
PLA: Proximity ligation assay
WT: Wildtype
KO: Knockout
NAb: Neutralizing antibody
